# Characterizing and quantifying disease impacts of generalized myasthenia gravis (gMG) in the United States: insights from patient and caregiver interviews and surveys

**DOI:** 10.1186/s41687-026-01124-y

**Published:** 2026-06-23

**Authors:** Pushpa Narayanaswami, Jamie Sullivan, Craig Thiele, Christina Ramirez, Susan dosReis, Allison Foss, Karen S. Yee, Mayvis Rebeira, Naila Wahid, Alexis C. Garduno, Elizabeth Crevier, Christine Rowe, Kelly G. Gwathmey

**Affiliations:** 1 Beth Israel Deaconess Medical Center/Harvard Medical School, Boston, USA; 2 EveryLife Foundation for Rare Diseases, Washington DC, USA; 3 Health Management Associates, Dayton, USA; 4 Patient Consultant, Rogers, USA; 5 University of Maryland School of Pharmacy, Baltimore, USA; 6 Myasthenia Gravis Association, Kansas City, USA; 7 Alexion, AstraZeneca Rare Disease, Boston, USA; 8 Avalere Health, Washington DC, USA; 9 Virginia Commonwealth University School of Medicine, Richmond, USA

**Keywords:** Myasthenia gravis, gMG, Disease burden, Interview, Survey, Caregiver

## Abstract

**Background:**

Treatment value is traditionally assessed through clinical benefits, health-related quality of life, and healthcare utilization. However, rare diseases such as generalized myasthenia gravis (gMG) that heavily affect families and/or caregivers require a more holistic approach that centers around impacted individuals across all areas of daily life. Here, key domains impacted by gMG were identified through individual interviews with both patients and caregivers and these impacts were further assessed through a survey in an independent sample.

**Methods:**

Interviews with patients with gMG and caregivers identified 25 patient-centered “impact elements” across 8 life domains (occupation, financial, emotional, physical, sleep, social, planning and autonomy, and safety). Independently recruited patients and caregivers who completed a subsequent electronic, quantitative survey rated the relevance of impact elements on a scale of 1 (lowest) to 5 (highest).

**Results:**

Respondents included 239 patients with gMG (63%, aged 18–49 y; 69%, female) and 81 caregivers (58%, aged 18–49 y; 42% female). The 5 domains most impacted by gMG were occupational (mean domain score [SD]: 4.0 [1.2]), planning and autonomy (4.0 [1.1]), financial (3.9 [1.2]), physical (3.9 [1.1]), and sleep (3.9 [1.2]) for patients and financial (3.7 [1.2]), sleep (3.7 [1.1]), occupational (3.6 [1.2]), planning and autonomy (3.6 [1.1]), and safety (3.6 [1.1]) for caregivers. Numerically higher impact scores were observed among patients with more severe disease, younger patients, and caregivers who were unemployed, retired, or disabled.

**Conclusions:**

These findings provide additional insight into the comprehensive burden of gMG that extends beyond symptoms and medical costs.

**Supplementary Information:**

The online version contains supplementary material available at 10.1186/s41687-026-01124-y.

## Background

Myasthenia gravis (MG) is a rare (estimated prevalence in the United States: 37 per 100,000), autoimmune neuromuscular disease characterized by fatigable muscle weakness [1–4]. Ocular weakness is the most common initial symptom of MG, occurring in 67%–85% of cases [5, 6]. Generalized MG (gMG) occurs when muscle weakness progresses beyond the eyes to include bulbar, limb, and/or axial muscle groups [5]. gMG develops within 2 years of symptom onset in 75%–85% of cases and this additional muscle weakness can lead to dysarthria, dysphagia, dyspnea, and fatigue [5–7]. Patients with gMG are also at risk of acute exacerbations (symptoms may include dysphagia, acute respiratory failure, or major functional disability that prevents physical activity) or a life-threatening myasthenic crisis (respiratory failure requiring endotracheal intubation and mechanical ventilation) [8, 9]. 

Several autoantibodies have been implicated in gMG pathophysiology, including those against acetylcholine receptor (AChR; approximately 85% of patients), muscle-specific tyrosine kinase (MuSK; 1%–10%), and low-density lipoprotein receptor-related protein 4 (LRP4; approximately 7% of patients without anti-AChR or anti-MuSK antibodies) [5, 10–14]; approximately 10% of cases are triple seronegative [15, 16]. Given the role of autoimmunity in gMG, routinely used therapies include steroidal and nonsteroidal immunosuppression, as well as thymectomy [17]. In recent years, several therapies that target and inhibit complement component 5 (C5) have been approved for acetylcholine receptor antibody–positive (AChR-Ab+) gMG [18–20]. 

The impact of rare diseases such as gMG is often defined by clinical outcomes of patients and direct medical costs borne by patients and payers [21, 22]. However, most studies do not address indirect costs to patients and their caregivers [21–23]. A study by the EveryLife Foundation estimated the total economic burden of rare disease in the United States in 2019 to be $997 billion, over half of which ($548 billion) resulted from indirect, nonmedical costs and noncovered healthcare costs [23]. The EveryLife Foundation analysis included costs from both patients with rare diseases and their caregivers, with caregiver costs accounting for nearly 40% ($205 billion) of indirect, nonmedical, and noncovered healthcare costs. Beyond economic costs, qualitative impacts are also crucial to evaluate. However, consequences such as the loss of health-related quality of life (HRQoL) for patients with rare disease and their caregivers are often overlooked [24]. When caregiver HRQoL is examined, it is often not evaluated directly but rather as a function of the patient’s health state or treatment [25]. 

Within gMG specifically, quantifying patient experiences can be challenging due to difficulty differentiating between drug-related effects and disease symptoms, symptom fluctuation and heterogeneity, and other confounding factors [1, 9, 26]. The fluctuating and unpredictable nature of gMG can negatively impact patients’ ability to perform activities of daily living, work productivity, mental health, quality of life, and economic well-being [1, 27, 28]. Despite these impacts, patient- and caregiver-provided data regarding their experiences with gMG and preferred treatment attributes remain limited [28, 29]. Therefore, there is a need to study the total disease impact to inform holistic, patient-centered value assessments, health insurance policy and coverage decisions, and clinical decision-making [21, 30]. 

The objective of the current analysis was to capture the multifaceted impacts of gMG and quantify the relative importance of key domains impacted by this disease through individual interviews with both patients and caregivers and to further assess these impacts through a survey in a larger, independent sample.

## Methods

### Interviews

Patients with physician-documented AChR-Ab+ gMG and caregivers who provided unpaid support participated in the semi-structured interviews. Caregivers were not required to provide antibody status documentation. Participants were recruited through a third-party firm (Sago, formerly Schlesinger Group).

**Fig. 1:**
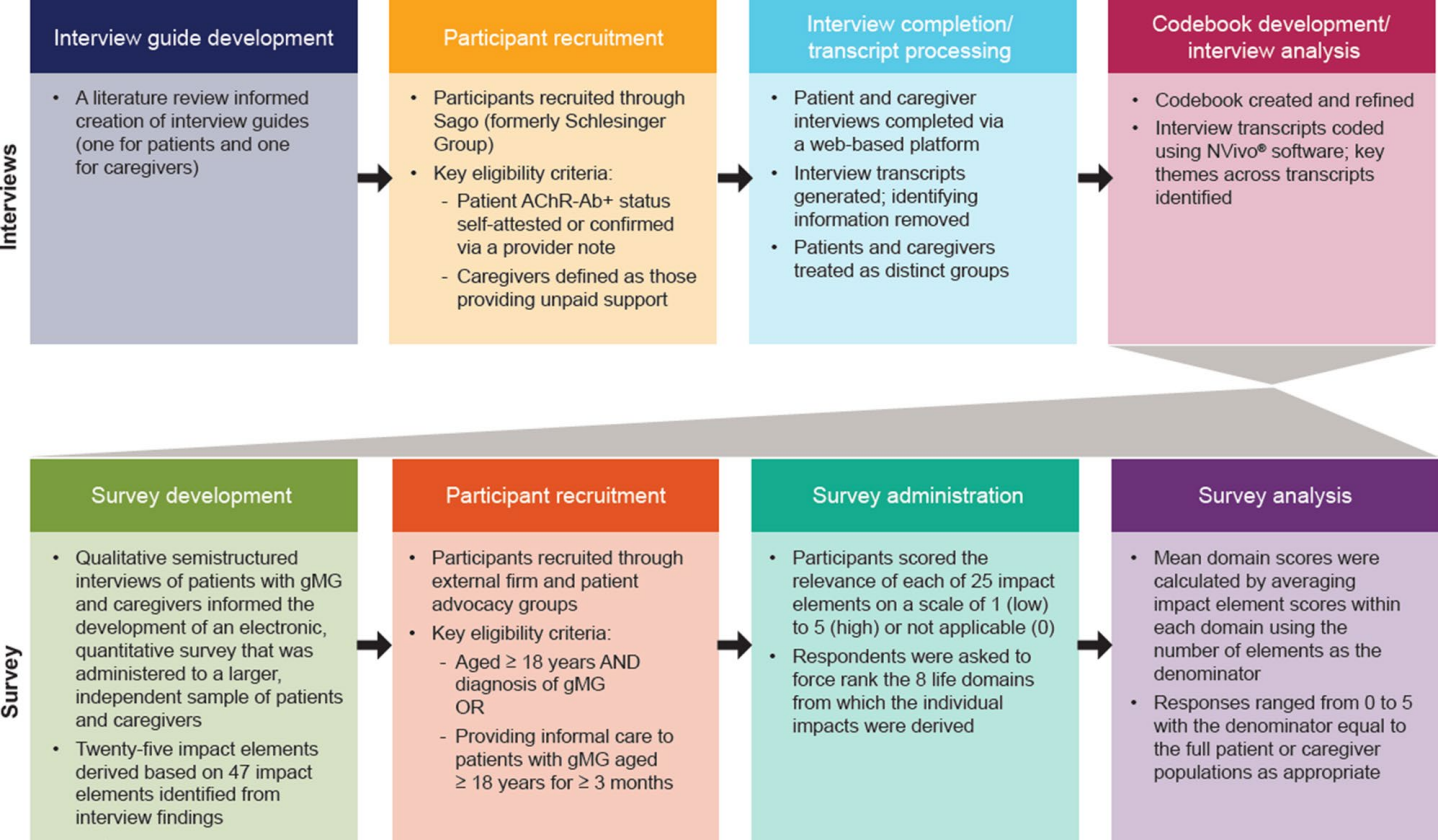
Study methodology. Abbreviations: AChR-Ab+ = anti-acetylcholine receptor antibody–positive, gMG = generalized myasthenia gravis

A literature review informed the creation of separate interview guides for patient and caregiver participants (see Supplementary Material for additional details). The guides were designed to elicit a deeper understanding of the impact of gMG on the life of the participant. Interviews were coded and qualitatively analyzed to summarize key points raised by participants (Fig. 1). Reported impacts not measured by the Myasthenia Gravis Activities of Daily Living (MG-ADL; validated patient-reported tool used to assess gMG symptom severity [31, 32]) or Myasthenia Gravis Quality of Life 15-item–Revised (MGQOL15r; validated patient-reported outcome measure assessing mobility, symptoms, general contentment, and emotional well-being [33]) scales were selected for further analysis through the survey. These impacts were classified in 3 hierarchical levels: impact experience (captures highest amount of detail, e.g., passing up career opportunities); impact element (captures multiple impact experiences, e.g., career aspirations); and domain (captures multiple impact elements, e.g., occupation).

Impacts were also classified as quantifiable (e.g., cost of services or supplies, direct or indirect medical costs, foregone income), possibly quantifiable (e.g., foregoing job opportunities, financial burden and stress resulting from medical costs [i.e., financial toxicity]), or nonquantifiable (e.g., emotional stress, disrupted work schedule). Classification of domains, impact elements, and impact experiences was approved by the study steering committee. Additionally, validation interviews and guided surveys with patients and caregivers were conducted to ensure the suitability and clarity of each domain and impact.

### Survey

Based on the interview findings, a set of impact elements and life domains was developed for use in the survey. The research team partnered with an external firm to identify, recruit, and enroll survey participants and collaborated with several gMG and rare disease patient and caregiver organizations (e.g., Myasthenia Gravis Foundation of America, Myasthenia Gravis Association, National Alliance for Caregiving, Facebook support groups) to publicize the survey. Potential participants were informed the study was funded by a life sciences company, but the specific sponsor was not revealed. Eligible patients were aged ≥ 18 years currently living in the United States with a self-reported physician diagnosis of gMG regardless of antibody status. Eligible caregivers were aged ≥ 18 years currently living in the United States and self-reported that they had been providing informal (e.g., unpaid, unskilled) care to a patient with gMG aged ≥ 18 years on a regular basis for ≥ 3 months. Formal caregiving support could not be the caregiver’s paid profession. To broaden access and ensure inclusion of a diverse sample, all participant-facing materials were translated into Spanish by a certified translator.

Respondents rated the relevance of each impact element on a scale of 0 (not applicable) to 5 (high) and ranked the 8 domains by importance and impact to their lives. Mean domain scores were calculated by averaging the impact element relevance scores within each domain. For subgroup analyses, impact element relevance scores were stratified by participant subgroup (i.e., patient or caregiver) and characteristics (Age: 18–49 years, 50–64 years, 65+ years; Sex: female, male; MG-ADL score: 0–6, 7–12, 13–18, 19–24).

### Analysis

Descriptive analyses included participant characteristics, such as sociodemographic, geographic, and key clinical characteristics. Continuous variables were summarized using the mean, median, and standard error. Categorical variables were summarized using the frequency (i.e., *n*) and relative frequency (i.e., %). No statistical testing was conducted.

## Results

### Patient and caregiver interviews – identifying impacts of gMG

Interviews were conducted July 19-September 1, 2022; all participants were based in the United States. Thirty interviews (17 patients and 13 caregivers) were completed using the respective interview guides, 28 of which (16 patients and 12 caregivers) met inclusion criteria and were included in the analysis (characteristics of interviewees are shown in Table 1). One patient interview was excluded from the study due to unknown AChR-Ab status, and 1 caregiver interview was deemed ineligible (paid caregiver).

**Table 1:** Participant demographics

Characteristic	Interviews		Surveys	
	Patients (*n* = 16)	Caregivers (*n* = 12)	Patients (*n* = 239)	Caregivers (*n* = 81)
**Has a caregiver**, ***n* (%)**	8 (50)	NA	169 (71)	NA
**Age**, ***n* (%)**				
18–49	9 (56)	7 (58)	150 (63)	47 (58)
50–64	7 (44)	4 (33)	65 (27)	32 (40)
65+	0	1 (8)	24 (10)	2 (2)
**Sex**, ***n* (%)**				
Female	13 (81)	7 (58)	165 (69)	34 (42)
Male	3 (19)	5 (42)	74 (31)	47 (58)
**Race**, ***n* (%)**				
American Indian or Alaska Native	1 (6)	0	0	2 (2)
Asian	1 (6)	0	5 (2)	5 (6)
Black or African American	6 (38)	4 (33)	20 (8)	7 (9)
White or Caucasian	7 (44)	7 (58)	206 (86)	64 (79)
Other/2 or more races	1 (6)	1 (8)	8 (3)	3 (4)
**Ethnicity**, ***n* (%)**				
Hispanic/Latino	0	2 (17)	27 (11)	12 (15)
Not Hispanic/Latino	16 (100)	10 (83)	212 (89)	69 (85)
**Time since diagnosis/caregiving began**, ***n* (%)**^**a**^				
Interviews				
≤ 5 years	5 (31)	6 (50)	-	-
> 5 years	11 (69)	6 (50)	-	-
Surveys				
≤ 2 years	-	-	69 (29)	26 (32)
> 2 years and ≤ 4 years	-	-	49 (21)	23 (28)
> 4 years and ≤ 10 years	-	-	76 (32)	25 (31)
More than 10 years	-	-	39 (16)	7 (9)
**MG-ADL score**, ***n* (%)**^**b**^				
0–6	-	-	68 (28)	-
7–12	-	-	115 (48)	-
13–18	-	-	51 (21)	-
19–24	-	-	5 (2)	-
**Antibody status**, ***n* (%)**^**a**^				
AChR-Ab+	16 (100)	12 (100)	181 (76)	60 (74)
MuSK-Ab+ only	0	0	8 (3)	2 (2)
LRP-4-Ab+ only	0	0	3 (1)	13 (16)
Seronegative	0	0	27 (11)	0
Unknown/not tested	0	0	0	6 (7)
More than 1 Ig+	0	0	20 (8)	0
**Primary insurance coverage**, ***n* (%)**				
Medicare fee-for-service	-	-	23 (10)	-
Medicare Advantage	-	-	27 (11)	-
Medicaid^c^	-	-	27 (11)	-
Commercial	-	-	145 (61)	-
Other	-	-	17 (7)	-

^a^For caregivers, time from diagnosis and antibody status were reported for the patient they cared for. ^b^MG-ADL score was not collected from interviewees. ^c^Includes Medicaid Managed Care

Abbreviations: AChR-Ab+ = anti-acetylcholine receptor antibody–positive, Ig+ = immunoglobulin positive, LRP-4-Ab+ = low-density lipoprotein receptor–related protein 4 antibody–positive, MG-ADL = Myasthenia Gravis Activities of Daily Living, MuSK-Ab+ = muscle-specific kinase antibody–positive, NA = not applicable

**Fig. 2:**
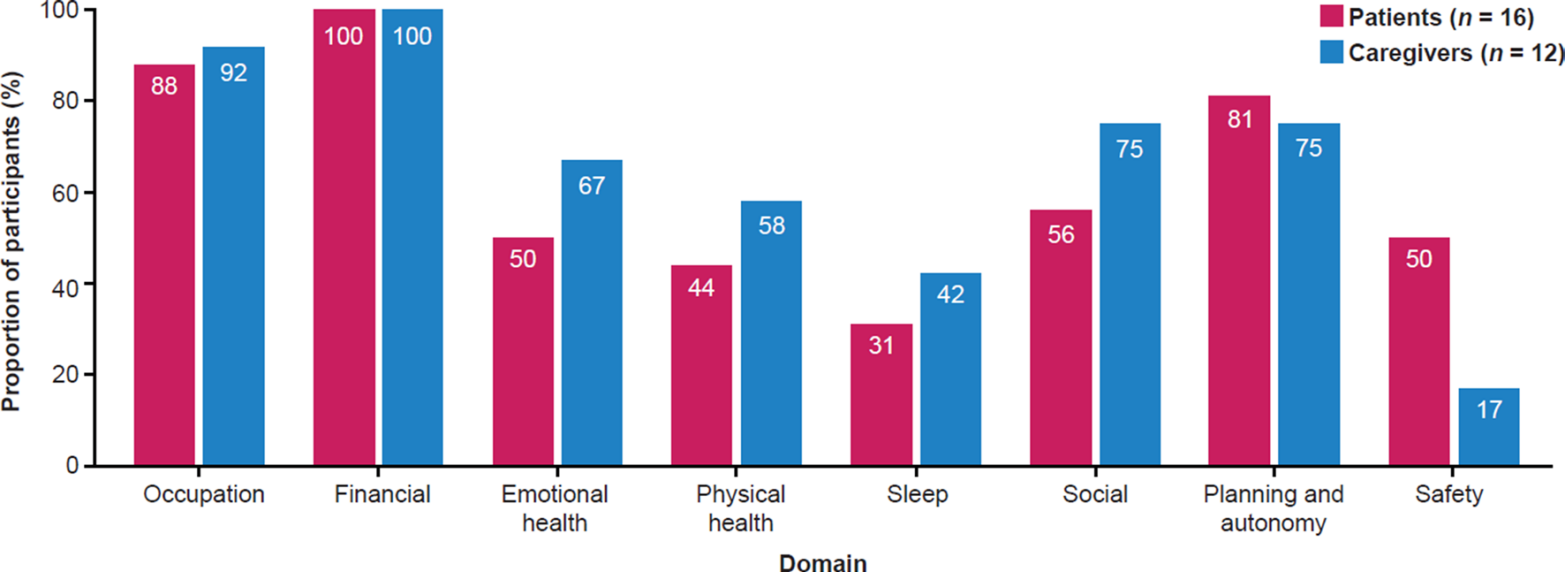
Impact domains reported by interview participants

A total of 84 impact experiences of gMG were identified and categorized into 25 impact elements across 8 domains (occupation, financial, emotional health, physical health, sleep, social, planning and autonomy, and safety), indicating effects on many aspects of patients’ and caregivers’ lives (For descriptions of all domains, impact elements, and impact experiences, see Supplementary Table 1). In 5 of the 8 domains (occupation, emotional health, physical health, sleep, social), a greater proportion of caregivers than patients reported impacts. More patients than caregivers reported impacts in the domains of planning and autonomy and safety. All interviewees reported financial impacts (Fig. 2). Patients and caregivers reported a mixture of quantifiable and nonquantifiable economic impacts, with the majority of impact experiences identified being economically nonquantifiable. The domains with the highest number of nonquantifiable impacts reported were planning and autonomy, emotional health, and social. The domains with the highest number of quantifiable impact experiences were financial (10 of 18) and occupation (5 of 14).

Of the 84 total impacts identified, 14 were not previously reported in the literature (Supplementary Table 1). Within the planning and autonomy domain, newly identified impacts included patients strategically budgeting their limited energy to prioritize their health when planning and caregivers feeling constantly on call:

- *“MG requires a lot of planning. The best way I describe it is I have an energy budget. I have to watch that budget and plan the budget because I can tell – I have a Fitbit and I have that Fitbit because I can tell if I did 25 thousand steps*,* I have to plan that next day to do nothing or I pay for 3 or 4 days if I don’t.”* -Patient 11
- *“You’re anticipating a call*,* if that makes sense. Whenever I see the phone rings and the call comes through*,* I’m like*,* “… I hope it’s just ‘don’t forget the oranges.’” You’re always walking on glass. […] You’re always alert.”* -Caregiver 10

Interviews revealed safety impact elements not cited in the literature, including expressing fear or powerlessness due to physical decline from gMG as well as safety concerns pertaining to treatment and the real or perceived medical mistreatment that can occur:

- *“A lot of times I don’t like walking with my cane because disabled people are often victims of crime. I live in a city with a lot of crime*,* so sometimes that’s in the back of my mind*,* because I can’t run*,* I can’t scream*,* because of the myasthenia gravis.”* -Patient 17
- *“…she’s had in-home care where we had a nurse come*,* and that was a little stressful because you didn’t really know if the nurse was able to do what they needed to do or not*,* because we had different ones. She’s had treatment that has landed her in the hospital because of the reactions*,* and so treatment has always been stressful.”* -Caregiver 2

In some domains, patients and caregivers were affected similarly, while in others they had more distinct experiences. For example, within the financial domain, both patients and caregivers noted that gMG-related needs resulted in increased financial burden due to high-cost expenses:

- *“It takes a lot of money to maintain this body…Having MG*,* it costs extra. I have to pay for convenience*,* food delivery*,* transportation services*,* medications that are not covered by insurance*,* that’s a substantial cost.”* -Patient 11
- *“We redid all the floors and leveled all the floors because it’s an old house*,* so it was a bit wobbly. We redid the back of the house*,* the access to the yard*,* because it was a few steps*,* so we increased the height of the deck so it’s flat from the house. We redid the bathroom so it’s ADA accessible. We still have a few things to do. Put a ramp in the front of the house.”* -Caregiver 5

In addition, both patients and caregivers reported withdrawing from social activities following the patient being diagnosed with gMG:

- *“I don’t really have a social life anymore. When I got MG*,* I was in college and I was very social*,* pretty active*,* active in our community*,* active around our school campus. After a couple of falls*,* it was like*,* not really wanting to fall in front of everyone*,* or get all the questions*,* the stares*,* the this*,* the that*,* and so a lot of that I just stopped.”* -Patient 10
- *“Before*,* we would do a lot of things with the kids. We used to take them out…But after diagnosis*,* all of that stopped. Everything just stopped.”* -Caregiver 7

Conversely, patients and caregivers reported differing effects of gMG on emotional health. For example, patients described how the strain of living with a chronic disease caused them to feel like their life revolves around their condition:

- *“But unfortunately*,* everything about me now is about this disease*,* which is really not who I am. So that’s been kind of a drag because everyone’s like*,* “oh*,* how are you?” It’s like well*,* you know.”* -Patient 1

Meanwhile, caregivers expressed feelings of guilt about how caring for someone with gMG impacts their own emotional well-being:

- *“I feel selfish talking about my emotional health*,* because I think of what she deals with*,* but I do have to acknowledge that it does impact my emotional health as well.”* -Caregiver 4
- *“Sometimes it takes over what I have to do for myself. I put myself last and put her first.”* -Caregiver 8

### Patient and caregiver survey – assessing impacts of gMG

Respondents to the survey included 239 patients with gMG and 81 caregivers (Table 1). Patients primarily had an informal caregiver, a MG-ADL score ≤ 12, and were receiving a variety of treatments. The majority of patients had commercial insurance as their primary coverage, with most having insurance from a single provider.

The most relevant domains for patients were financial, planning and autonomy, and physical (Table 2). For caregivers, the most relevant domains were financial, sleep, and planning and autonomy. The financial and physical domains were also most frequently ranked as the most impactful by both patients and caregivers (Supplementary Table 2, Supplementary Table 3).

**Table 2:** Summary of mean domain relevance scores from survey respondents

Domain	Relevance score, mean (SD)^a^	
	Patient	Caregiver
Financial	3.8 (1.4)	3.6 (1.4)
Planning and autonomy	3.8 (1.3)	3.5 (1.1)
Physical	3.7 (1.3)	3.4 (1.2)
Sleep	3.4 (1.7)	3.5 (1.3)
Social	3.4 (1.6)	3.3 (1.3)
Occupational	3.3 (1.8)	3.3 (1.6)
Safety	3.3 (1.6)	3.2 (1.5)
Emotional	3.3 (1.5)	3.1 (1.5)

Abbreviation: SD = standard deviation

^a^Among subgroups, relevance scores of the 25 impact elements were generally higher for patients with more severe disease (vs. less severe), younger patients (vs. older), and female patients (vs. male) (Fig. 3). Impact element relevance scores were higher for caregivers who were unemployed, retired, or disabled (vs. caregivers who were employed full-time) and female caregivers (vs. male) (Fig. 4)

**Fig. 3:**
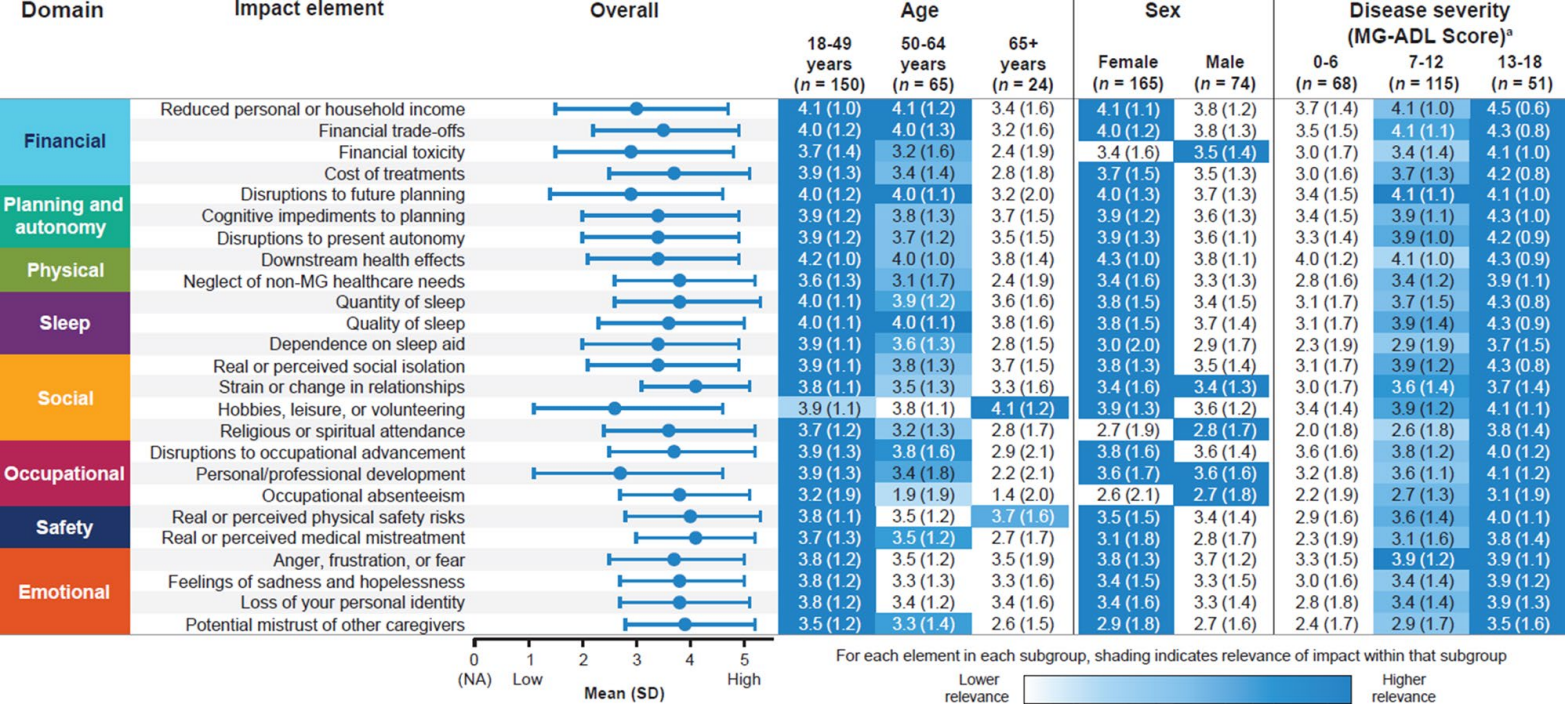
Patient relevance of impact elements by subgroups. ^a^The MG-ADL score 19–24 subgroup (*n* = 5) was not included on the basis of small sample size. Abbreviations: MG = myasthenia gravis, MG-ADL = Myasthenia Gravis Activities of Daily Living, NA = not applicable

**Fig. 4:**
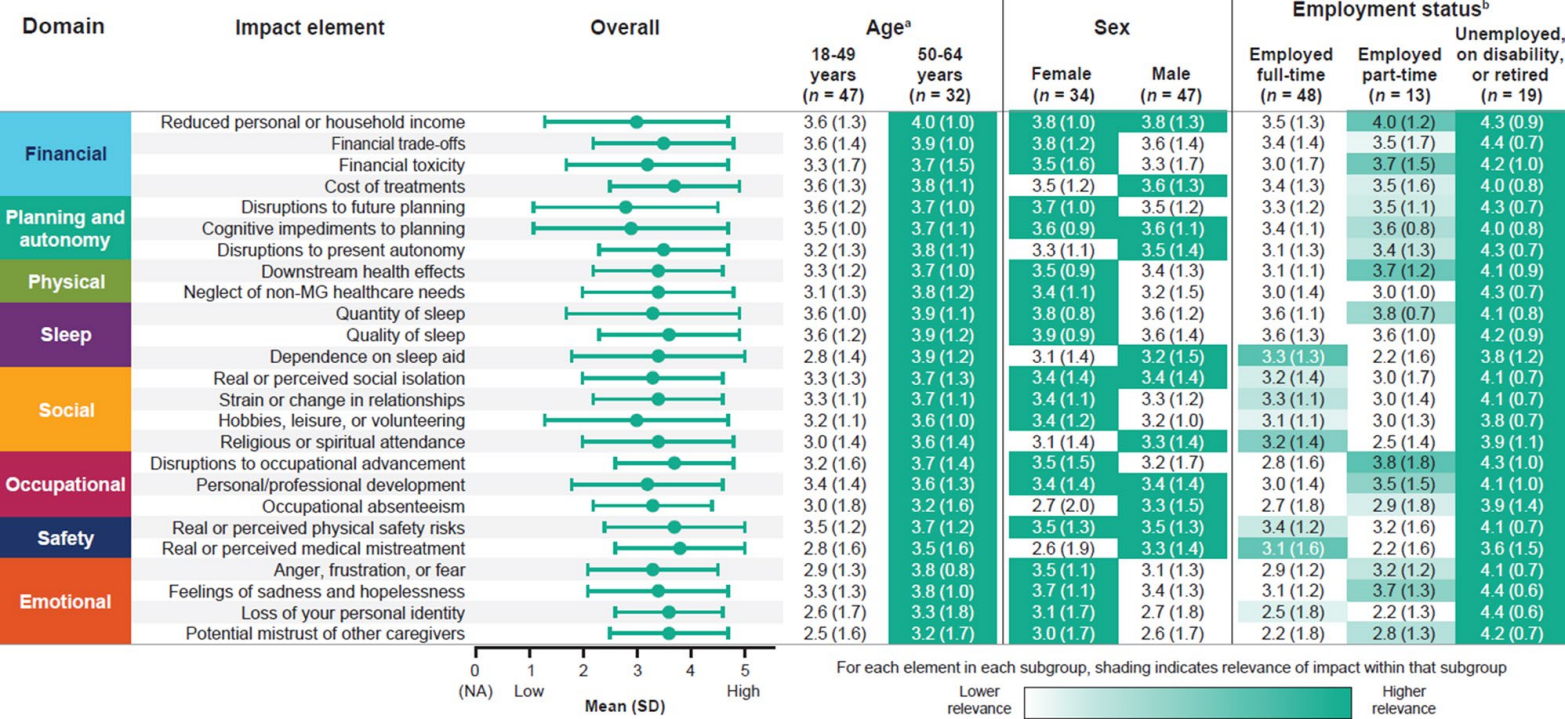
Caregiver relevance of impact elements by subgroups. ^a^The aged 65+ years (*n* = 2) subgroup was not included on the basis of small sample size. ^b^The seeking work or in school (*n* = 1) subgroup was not included on the basis of small sample size. Abbreviations: MG = myasthenia gravis, NA = not applicable

## Discussion

Understanding the holistic impact of gMG for patients and their caregivers can provide payers, policymakers, and healthcare professionals (HCPs) with important information to consider when making decisions that can affect patient care and services. This study aimed to thoroughly examine gMG-related burdens, including aspects not measured in current disease-related instruments, and assess their relative importance. First, qualitative semi-structured interviews of patients and caregivers were conducted to inform a more comprehensive framework of the impact of gMG. In a subsequent survey developed based on the interviews, mean relevance scores were high and similar across the 8 domains in the survey population. In addition, relevance scores were similar across the 25 impact elements surveyed with the majority of relevance scores for both patients and caregivers being ≥ 3. This suggests domains and impact elements identified through the interviews are salient features of the overall true gMG burden.

Patients tended to assign numerically higher domain scores relative to caregivers. The largest differences in relevance scores between patients and caregivers were in the physical and planning and autonomy domains. The difference between the 2 groups was driven by the high relevance patients placed on “neglect non-MG healthcare needs.” Although it is reasonable to deduce that people with gMG would find the physical domain more relevant as they are more likely to suffer physical health consequences as a direct result of their disease, caregivers likely experience negative physical health consequences as well. A cross-sectional study of caregiver burden in 5 European countries found that physical health scores of caregivers for individuals with MG were significantly lower than the general population, and 34% of caregivers were unable to carry out everyday physical activities [34]. These findings underscore the need to address all aspects of physical health for both patients and caregivers.

Within the planning and autonomy domain, the higher relevance scores from patients for impact elements such as “disruptions to present autonomy” may reflect their direct experience with gMG-related limitations. This is consistent with previous work finding that patients and caregivers often feel like they are losing independence or control in their lives [34, 35]. Impact elements within the planning and autonomy domain are not easily quantifiable, further illustrating how traditional economic analyses underestimate the total burden of gMG. Future research should investigate potential within- and between-group differences in a larger sample.

The financial domain had the highest numerical relevance score and was among the 2 most impactful domains for both patients and caregivers. The “reduced personal/household income” and “making financial trade-offs” impact elements were the largest contributors. The degree of relevance placed on these impact elements, as well as others relative to the “cost of treatments” relevance score, suggests that indirect costs and impacts that are difficult to monetize are at least as relevant to patient and caregiver lives as direct medical costs. The importance of indirect costs is also evidenced by relevance scores of the impact elements in the occupational domain. “Disruptions to occupational advancement” and “personal/professional development,” were scored directionally higher than “occupational absenteeism.” Although the latter element is often captured in traditional economic burden analyses [36, 37], our findings suggest a large portion of the occupational impact of gMG is not accounted for, as disruptions to occupational advancement and professional development cannot be quantified with a monetary value and thus less likely to be included in such analyses.

Despite the emotional, social, safety, and sleep domains being ranked lower, impact elements such as sadness and depression or poor sleep quality likely have implications for patient and caregiver quality of life even if individuals are not directly aware of the impacts. Consistent with this idea, a qualitative analysis of semi-structured interviews found that HCPs often identify anxiety and depression symptoms in their patients with MG even when patients do not directly express mental health concerns [35]. Furthermore, real or perceived medical mistreatment or physical safety risks could cause a delay in seeking care and prolong recovery. Additional insights into these impact elements may aid understanding of patients’ and caregivers’ experiences.

The numerically higher impact scores observed among patients with greater disease severity and older age are consistent with previous work showing these factors can negatively affect quality of life and physical health in patients with MG or gMG [38–40]. However, to our knowledge, caregiver characteristics affecting the relevance of gMG in their lives have not been explored elsewhere. Higher survey relevance scores for unemployed caregivers could reflect that they may be less equipped to handle the financial challenges of caring for someone with gMG. Nevertheless, more work is needed to ascertain the underlying causes of these differences.

Although it is important to consider these impact elements separately, it is possible that some are interconnected. For instance, poor sleep quality could lead to occupational absenteeism and/or loss of productivity, suggesting that impact elements that cannot be directly monetized may still have substantial economic consequences. The extensive impacts of gMG on both patients and caregivers illustrate the breadth of disease burden that is not fully captured by traditional economic and quality-of-life analyses. Thus, a more comprehensive understanding of the burden of gMG is necessary to improve patient outcomes.

A recent needs assessment found that while patients and HCPs were generally aligned in their knowledge of MG symptomatology, there were differences in how the impact of these symptoms was perceived [35]. Patients expressed greater concern for how their symptoms affect their daily lives and how they are perceived by others, while HCPs were focused on managing the risk of hospitalization and preventing myasthenic crises [35]. The current findings will help HCPs better understand how patients and caregivers perceive the relevance and impact of gMG symptoms, which may inform improved management strategies and treatment selection.

Limitations of this work include the small interview sample, which may not accurately capture the diversity of patients with AChR-Ab+ gMG in the United States. Caregiver interviewees were not required to verify the antibody status of the patient from whom they provide care. It is possible caregivers were not aware of or misreported the patient’s antibody status. Full patient medical history was not collected during the interview or survey. For instance, severity of gMG, existing comorbidities such as depression, and concomitant medications were not captured. Additional limitations of the survey include possible bias arising from self-selection of participants and the potential for sampling and recall bias. The small sample sizes did not justify statistical testing, and future work with a larger sample could allow for more generalizable insights. In addition, because this study aimed to benchmark the holistic burden of disease, both analyses were cross-sectional and treatment-agnostic by design. Although the longitudinal effects and influence of specific therapies on impact domains and elements cannot be evaluated here, the current approach provides a comprehensive foundation that can inform future treatment-specific burden reduction goals across different therapeutic interventions.

## Conclusions

gMG imposes a substantial burden on patients and caregivers across a broad range of life domains, including impacts on finances, physical health, and planning and autonomy. These impacts, many of which cannot be quantified with a monetary value, should be considered when assessing the overall disease burden of gMG in addition to direct medical costs, patient outcomes, and treatment benefits. Future evaluation of these impacts will be important to better understand the potential benefits associated with gMG treatments. Impact elements identified for the gMG population in this study are also relevant for other rare diseases. Promoting standards for measuring these factors is likely to benefit gMG and other rare disease research.

## Supplementary Information

Below is the link to the electronic supplementary material.

Supplementary Material 1

## Data Availability

Alexion, AstraZeneca Rare Disease, will consider requests for disclosure of clinical study participant-level data provided that participant privacy is assured through methods like data de-identification, pseudonymization, or anonymization (as required by applicable law), and if such disclosure was included in the relevant study informed consent form or similar documentation. Qualified academic investigators may request participant-level clinical data and supporting documents (statistical analysis plan and protocol) pertaining to Alexion-sponsored studies. Further details regarding data availability and instructions for requesting information are available in the Alexion Clinical Trials Disclosure and Transparency Policy at https://www.alexionclinicaltrialtransparency.com/data-requests.

## Abbreviations

AChR: Acetylcholine receptor
AChR-Ab+: Acetylcholine receptor antibody–positive
C5: Complement component 5
gMG: Generalized myasthenia gravis
HCP: Healthcare professional
HRQoL: Health-related quality of life
Ig+: Immunoglobulin positive
LRP-4: Low-density lipoprotein receptor–related protein 4
LRP-4-Ab+: Low-density lipoprotein receptor–related protein 4 antibody–positive
MG: Myasthenia gravis
MG-ADL: Myasthenia Gravis Activities of Daily Living
MGQOL15r: Myasthenia Gravis Quality of Life 15-item–Revised
MuSK: Muscle-specific kinase
MuSK-Ab+: Muscle-specific kinase antibody–positive
NA: Not applicable
SD: Standard deviation
