# Supplementary material for: Characterizing and quantifying disease impacts of generalized myasthenia gravis (gMG) in the United States: insights from patient and caregiver interviews and surveys
Source: J Patient Rep Outcomes. 2026 Jun 23;10:126. doi: 10.1186/s41687-026-01124-y (PMC13400517; doi:10.1186/s41687-026-01124-y)
Supplement: Supplementary file 1 — Supplementary Material 1 [file 41687_2026_1124_MOESM1_ESM.docx]

**Supplementary Material**

## **Literature review**

The literature review consisted of a targeted PubMed search of results published between March 1, 2002, and March 1, 2022, a targeted gray literature search conducted in April 2022, and manual addition of publications shared by the study sponsor. The PubMed search strings included “myasthenia gravis [MeSH Terms]” paired separately with each of the following terms using the AND operator**:** patient treatment preference; patient voice; initial consultation; navigation of referral; barriers; management of referral; patient preference OR caregiver preference OR caregiver; newly diagnosed; goals; decision making; lifestyle; preference; provider discussion; challenges; patient needs; follow-up; care coordination; management [MeSH Terms]; treatment [MeSH Terms]; diagnosis [MeSH Terms]; quality of life [MeSH Terms]; referral [MeSH Terms]; daily life [MeSH Terms]; care delivery [MeSH Terms].

Gray literature and other internet sources included the American Academy of Neurology, Conquer MG, U.S. Food and Drug Administration, MG Foundation of America, National Health Council, National Organization for Rare Diseases, and RareConnect.

Excluded publication types were animal studies, case reports, clinical trial protocols, comments, datasets, editorials, interviews, news, lectures, legal cases, legislation, letters, periodical indices, personal narratives, research support, scientific integrity reviews, and webcasts. Non-English abstracts and studies in participants <18 years of age were also excluded.

The literature screening process is summarized in Supplementary Figure 1 below:

**Supplementary Figure 1.** Evidence identification and selection process.


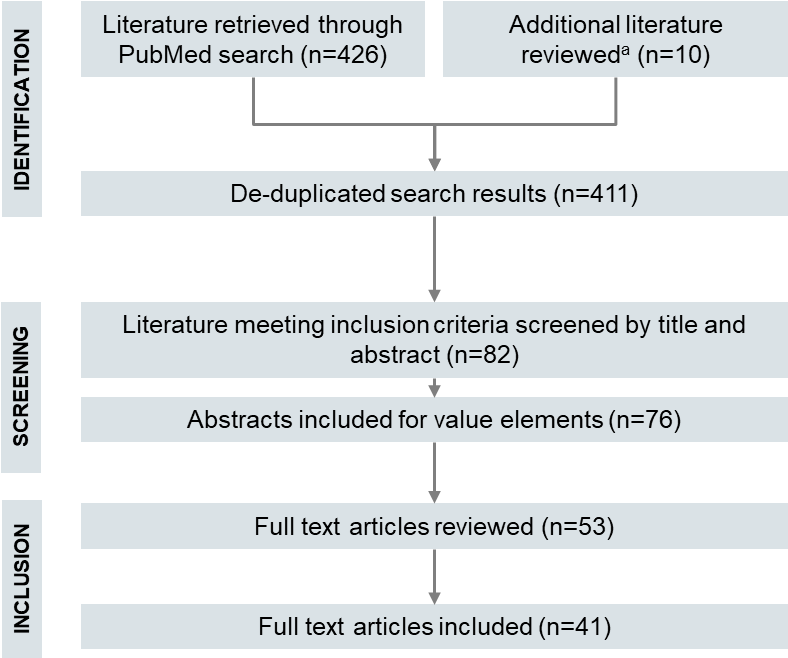

^a^Includes 7 references suggested by the study sponsor.

The full text articles (listed below) were used to identify potential impacts and inform the development of the interview guides.

- Alqarni F, Almalki D, Aljohani Z, et al. Prevalence and risk factors of myasthenia gravis recurrence post-thymectomy. *Neurosciences (Riyadh)*. 2021;26(1):4-14.
- Angelis A, Kanavos P. Multiple criteria decision analysis (MCDA) for evaluating new medicines in health technology assessment and beyond: the advance value framework. *Soc Sci Med*. 2017;188:137-156.
- Angelis A, Linch M, Montibeller G, et al. Multiple criteria decision analysis for HTA across four EU member states: piloting the advance value framework. *Soc Sci Med*. 2020;246:112595.
- Angelis, A., Kanavos, P., Phillips, LD. ICER Value Framework 2020 Update: recommendations on the aggregation of benefits and contextual considerations. *Value Health*. 2020;23(8):1040-1048.
- Angelis A, Kanavos P. Value-based assessment of new medical technologies: towards a robust methodological framework for the application of multiple criteria decision analysis in the context of health technology assessment. *Pharmacoeconomics*. 2016;34(5):435-446.
- Barnett C, Bril V, Kapral M, Kulkarni A, Davis AM. A conceptual framework for evaluating impairments in myasthenia gravis. *PLoS One*. 2014;9(5):e98089.
- Barnett C, Herbelin L, Dimachkie MM, Barohn RJ. Measuring clinical treatment response in myasthenia gravis. *Neurol Clin*. 2018;36(2):339-353.
- Berrih-Aknin S, Claeys KG, Law N, et al. Patient-reported impact of myasthenia gravis in the real world: protocol for a digital observational study (MyRealWorld MG). *BMJ Open*. 2021;11(7):e048198.
- Boldingh MI, Dekker L, Maniaol AH, et al. An update on health-related quality of life in myasthenia gravis -results from population-based cohorts. *Health Qual Life Outcomes*. 2015;13:115.
- Cavaller-Bellaubi M, Faulkner SD, Teixeira B, et al. Sustaining meaningful patient engagement across the lifecycle of medicines: a roadmap for action. *Ther Innov Regul Sci*. 2021;55(5):936-953.
- Cherukupally KR, Kodjo K, Ogunsakin O, Olayinka O, Fouron P. Comorbid depressive and anxiety symptoms in a patient with myasthenia gravis. *Case Rep Psychiatry*. 2020;2020:8967818.
- Costamagna G, Abati E, Bresolin N, Comi GP, Corti S. Management of patients with neuromuscular disorders at the time of the SARS-CoV-2 pandemic. *J Neurol*. 2021;268(5):1580-1591.
- de Souza JA, Seiwert TY. A value framework in head and neck cancer care. *Am Soc Clin Oncol Educ Book*. 2014;e304-e309.
- dosReis S, Butler B, Caicedo J, et al. Stakeholder-engaged derivation of patient-informed value elements. *Patient*. 2020;13(5):611-621.
- Erşen E, Kılıç B, Kara HV, et al. Comparative study of video-assisted thoracoscopic surgery versus open thymectomy for thymoma and myasthenia gravis. *Wideochir Inne Tech Maloinwazyjne*. 2018;13(3):376-382.
- Faulkner SD, Sayuri Ii S, Pakarinen C, et al. Understanding multi-stakeholder needs, preferences and expectations to define effective practices and processes of patient engagement in medicine development: a mixed-methods study. *Health Expect*. 2021;24(2):601-616.
- U.S. Food and Drug Administration. Patient-focused drug development: methods to identify what is important to patients. Guidance for industry, Food and Drug Administration staff, and other stakeholders. Feb 2022. <https://www.fda.gov/media/131230/download>.
- Gagnon MP, Desmartis M, Gagnon J, et al. Introducing the patient's perspective in hospital health technology assessment (HTA): the views of HTA producers, hospital managers and patients. *Health Expect*. 2014;17(6):888-900.
- Goetghebeur M, Cellier M. Deliberative processes by health technology assessment agencies: a reflection on legitimacy, values and patient and public involvement comment on "Use of Evidence-informed Deliberative Processes by Health Technology Assessment Agencies Around the Globe". *Int J Health Policy Manag*. 2021;10(4):228-231.
- Gunn CJ, Bertelsen N, Regeer BJ, Schuitmaker-Warnaar TJ. Valuing patient engagement: reflexive learning in evidence generation practices for health technology assessment. *Soc Sci Med*. 2021;280:114048.
- Hashem F, Calnan MW, Brown PR. Decision making in NICE single technological appraisals: How does NICE incorporate patient perspectives? *Health Expect*. 2018;21(1):128-137.
- Huls SPI, Whichello CL, van Exel J, Uyl-de Groot CA, de Bekker-Grob EW. What is next for patient preferences in health technology assessment? A systematic review of the challenges. *Value Health*. 2019;22(11):1318-1328.
- ICER. 2020-2023 Value Assessment Framework. Oct 23, 2020. <https://icer.org/wp-content/uploads/2020/10/ICER_2020_2023_VAF_102220.pdf>
- Janssens R, Huys I, van Overbeeke E, et al. Opportunities and challenges for the inclusion of patient preferences in the medical product life cycle: a systematic review. *BMC Med Inform Decis Mak*. 2019;19(1):189.
- Janssens R, Russo S, van Overbeeke E, et al. Patient preferences in the medical product life cycle: what do stakeholders think? Semi-structured qualitative interviews in Europe and the USA. *Patient*. 2019;12(5):513-526.
- Jeong A, Min JH, Kang YK, et al. Factors associated with quality of life of people with myasthenia gravis. *PLoS One*. 2018;13(11):e0206754.
- Lakdawalla DN, Phelps CE. Health technology assessment with diminishing returns to health: the generalized risk-adjusted cost-effectiveness (GRACE) approach. *Value Health*. 2021;24(2):244-249.
- Masi D, Gomez-Rexrode AE, Bardin R, Seidman J. The "Preparation for Shared Decision-Making" tool for women with advanced breast cancer: qualitative validation study. *J Particip Med*. 2019;11(4):e16511.
- Mercer RE, Chambers A, Mai H, McDonald V, McMahon C, Chan KKW. Are we making a difference? A qualitative study of patient engagement at the pan-Canadian Oncology Drug Review: perspectives of patient groups. *Value Health*. 2020;23(9):1157-1162.
- Nagane Y, Murai H, Imai T, et al. Social disadvantages associated with myasthenia gravis and its treatment: a multicentre cross-sectional study. *BMJ Open*. 2017;7(2):e013278.
- Neumann PJ, Garrison LP, Willke RJ. The history and future of the "ISPOR Value Flower": addressing limitations of conventional cost-effectiveness analysis. *Value Health*. 2022;25(4):558-565.
- Pearson SD. The ICER Value Framework: integrating cost effectiveness and affordability in the assessment of health care value. *Value Health*. 2018;21(3):258-265.
- Peres J, Martins R, Alves JD, Valverde A. Rituximab in generalized myasthenia gravis: clinical, quality of life and cost-utility analysis. *Porto Biomed J*. 2017;2(3):81-85.
- Salari N, Fatahi B, Bartina Y, et al. Global prevalence of myasthenia gravis and the effectiveness of common drugs in its treatment: a systematic review and meta-analysis. *J Transl Med*. 2021;19(1):516.
- Shafrin J, Schwartz TT, Okoro T, Romley JA. Patient versus physician valuation of durable survival gains: implications for value framework assessments. *Value Health*. 2017;20(2):217-223.
- Szczudlik P, Sobieszczuk E, Szyluk B, Lipowska M, Kubiszewska J, Kostera-Pruszczyk A. Determinants of quality of life in myasthenia gravis patients. *Front Neurol*. 2020;11:553626.
- Thaker NG, Pugh TJ, Mahmood U, et al. Defining the value framework for prostate brachytherapy using patient-centered outcome metrics and time-driven activity-based costing. *Brachytherapy*. 2016;15(3):274-282.
- Twork S, Wiesmeth S, Klewer J, Pöhlau D, Kugler J. Quality of life and life circumstances in German myasthenia gravis patients. *Health Qual Life Outcomes*. 2010;8:129.
- van Overbeeke E, Forrester V, Simoens S, Huys I. Use of patient preferences in health technology assessment: perspectives of Canadian, Belgian and German HTA representatives. *Patient*. 2021;14(1):119-128.
- Whittal A, Meregaglia M, Nicod E. The use of patient-reported outcome measures in rare diseases and implications for health technology assessment. *Patient*. 2021;14(5):485-503.
- Willke RJ, Neumann PJ, Garrison LP Jr, Ramsey SD. Review of recent US value frameworks-a health economics approach: an ISPOR special task force report [6]. *Value Health*. 2018;21(2):155-160.

## **Supplementary Table 1** Impacts identified in the patient and caregiver interviews.

| Domains (total: 8) | Impact elements **+11 new** (total: 47) | Impact experiences **+14 new** (total: 84) |
| --- | --- | --- |
| Financial | Reduced personal or household income | - Fixed income - Lack of savings - Taking on additional dependents (caregiver only) - Increased responsibility for income as sole provider (caregiver only) - Needed income to support cost of living outpacing raises |
|  | Financial tradeoffs | - Reduced discretionary income/“fun” money - Need to defer big purchases (eg, house) |
|  | Financial toxicity | - Skipping doses or not filling prescriptions (patient only) - Choosing less effective treatment options (patient only) - Reliance on high-risk external financial support (eg, loans; patient only) - Reliance on charitable giving (eg, patient support programs, grants; patient only) - Financial stress - Reduced spending on necessities (eg, food, clothing, utilities) |
|  | OOP costs | - High cost of gMG treatments directly related to gMG care |
|  | **Cost of ADL needs** | - Increased expenses to support daily needs (eg, gas, transportation, parking) - **Need to hire household support (eg, babysitter, cleaners; patient only)** |
|  | **High-cost expenses to support quality of life** | - **Increased high-cost expenses (eg, car, medical devices, home modifications)** - Reside in high cost-of-living areas (near specialists) |
| Planning and autonomy | **Vigilance** | - **Hypervigilance, including frequent or constant alertness, expecting the worst** - **Overplanning, including logistical coordination, preparing for the worst** - Awareness/caution of sensitivity to environmental triggers and communicable diseases (eg, heat, COVID, or other contagions, stairs, accessibility) |
|  | Disruptions to plans | - Forgoing, delaying, shortening, or generally changing planned activities; includes inability to plan/having to be reactive to situations (reactivity) |
|  | **Autonomy** | - Lack of choice in living situation, including where to live, what kind of dwelling to live in, living arrangements - **Altering priorities** - Reduced independence (patient only) |
|  | Instability | - Uncertainty about the future, not knowing what each day will bring (patient only) |
|  | Personal aspirations | - Reduced ability to set personal goals (being a mother, being a spouse, living on a farm, etc.) - Changes to previously set life goals and aspirations |
|  | **Life adaptations** | - Simplifying routines, such as cooking simpler meals, buying in smaller quantities that can be more easily held (patient only) - **Budgeting of energy/time to have enough for activities (patient only)** |
|  | Illness work | - Time spent managing healthcare system (eg, insurance, medical providers, and other healthcare needs); often takes time away from other aspects of life |
| Physical health | Neglecting health needs | - Neglecting non-MG healthcare needs - Reduced focus on personal health and wellness |
|  | Downstream health impacts | - Reduced functionality - MG-related comorbidities - Fatigue/exhaustion - Headaches - Weight changes |
| Sleep | Insomnia | - Unable to sleep, staying up all night, etc. |
|  | Quality of sleep | - Irregular sleep schedule (eg, going to sleep late due to lack of time, taking naps to make up for exhaustion, poor sleep quality)​ - Not sleeping comfortably/well; may be due to stress, physical discomfort, disruption from devices, etc.)​ - Waking up frequently (eg, sleeping with one eye open, constantly alert, shallow sleep) |
|  | **Reliance on sleep aids** | - **Need to use sleep-promoting medication to sleep** |
| Social | Strain or change to relationships—Intimate | - Negative impacts to intimate relationships and dating, including more distance, less connection, inability to see each other/make time for intimate relationships |
|  | Strain or change to relationships—Immediate | - Strain on family and friendships, lack of family understanding of MG, reduced quality time with immediate family or friends, difficult or strained communication with person with MG |
|  | Strain or change to relationships—Non-immediate | - Difficulty forming new friendships, includes more “limited social circle” |
|  | Social isolation | - Social isolation, wanting to stay at home, limit exposure to people and relationships |
|  | Leisure | - Reduced ability or desire to participate in social activities or events, including dinners, concerts, movies, etc. |
|  | Hobbies | - Reduced/lack of ability to participate in hobbies, including sports, crafts, and other pastimes |
|  | Volunteering | - Inability to volunteer, shift in volunteer activities or hours |
|  | Religious services | - Reduction in ability to attend religious services |
|  | Real or perceived negative public perception and poor public understanding | - Discomfort explaining situation to others, people don't understand, fear of and experienced social stigma, real or perceived mistrust or doubt of person/symptomatic experience |
| Occupation | Career aspirations | - Passing on job opportunities - Forego career or dream job - Career dissatisfaction |
|  | **Freedom of occupation** | - Having to change jobs to accommodate gMG needs - **Being “stuck” in a role (patient only)** |
|  | Unemployment or underemployment | - Unable to work (exited workforce; patient only) |
|  | **On-the-job disruption** | - Disrupted or irregular work schedule​ - **Require unique work arrangements​** - Unable to perform duties/responsibilities (patient only) |
|  | Productivity and performance | - Real or perceived reduced personal performance​ - Real or perceived reduced business performance |
|  | Absenteeism | - Paid leave (PTO, FMLA) - Unpaid leave |
|  | Educational disruption | - Defer or delay education |
| Safety | Real or perceived physical safety risks | - Fear and real dangers of driving (patient only) - Risk and experience of infections (eg, post-op; patient only) - Risk and experience of choking or breathing obstruction (patient only) - Risk and experience of falls (patient only) - Risk and experience of injuries |
|  | **Real or perceived medical mistreatment** | - **Greater risk of experiencing malpractice or medical mistreatment​** - **Worse outcomes when malpractice or medical mistreatment is experienced (patient only)** |
|  | **Powerlessness** | - **Vulnerability to crime; not being able to defend themselves (patient only)** |
| Emotional health | Anger or resentment | - Charged negative feelings that include outrage, denial, feeling misunderstood by care team, feeling indignant (caregiver only) |
|  | Fear or anxiety | - Fears, apprehensions, and other feelings of general anxiety, uncertainty of future; inclusive of treatment-related fear (eg, effectiveness, availability, coverage, risk of changing treatment), fear of injury |
|  | Frustration | - Feeling drained, dissatisfaction with care team's gMG awareness, dissatisfaction with performing day-to-day activities or inability to plan; includes experience of disconnect between body and brain |
|  | Sadness and depression | - Feelings of sorrow, feeling resigned, feeling depressed, negative thought spirals, giving up; includes reduced feelings of enjoyment and comments about "being drained" |
|  | Shame or embarrassment | - Inclusive of feeling like a burden, lack of self-esteem, poor body image |
|  | **Guilt** | - **Includes feeling selfish for talking about self/reflecting negatively on patient; guilt over being healthy** |
|  | Stress | - Inclusive of stress-induced insomnia, stress due to underperformance at work, etc. |
|  | **Loss of identity** | - **Personal identity is secondary or tied up in caregiving or having gMG (patient only)** |
|  | Impaired cognitive function | - Difficulty remembering, concentrating, making decisions, and learning new things; inclusive of inability to complete ADLs, job (patient only) |
|  | Disproportionate sense of responsibility | - Mistrust of other caregivers, expanded sphere/downstream impact on non-gMG family members; includes the need to protect the MG patient's sense of normalcy, dignity (caregiver only) |

Novel impacts not previously reported in the literature are shown in **bold text.**
Abbreviations: ADL = activities of daily living, FMLA = Family and Medical Leave Act, gMG = generalized myasthenia gravis, OOP = out-of-pocket, PTO = paid time off.

## **Supplementary Table 2** Patient rankings of impact domains

| **Rank** | **Domain (n, %)** | | | | | | | |
| --- | --- | --- | --- | --- | --- | --- | --- | --- |
|  | **Emotional health** | **Financial** | **Physical health** | **Planning and autonomy** | **Occupational** | **Safety** | **Sleep** | **Social** |
| 1 (most impact) | 35 (14.6%) | **50 (20.9%)** | **89 (37.2%)** | 16 (6.7%) | 17 (7.1%) | 11 (4.6%) | 14 (5.9%) | 7 (2.9%) |
| 2 | 43 (18.0%) | 33 (13.8%) | 61 (25.5%) | 23 (9.6%) | 32 (13.4%) | 18 (7.5%) | 17 (7.1%) | 12 (5.0%) |
| 3 | **47 (19.7%)** | 32 (13.4%) | 18 (7.5%) | 28 (11.7%) | 38 (15.9%) | 31 (13.0%) | 27 (11.3%) | 18 (7.5%) |
| 4 | 36 (15.1%) | 27 (11.3%) | 27 (11.3%) | 34 (14.2%) | 28 (11.7%) | 33 (13.8%) | 29 (12.1%) | 25 (10.5%) |
| 5 | 27 (11.3%) | 26 (10.9%) | 18 (7.5%) | 27 (11.3%) | 27 (11.3%) | 30 (12.6%) | **43 (18.0%)** | 41 (17.2%) |
| 6 | 19 (7.9%) | 26 (10.9%) | 16 (6.7%) | 36 (15.1%) | 28 (11.7%) | **43 (18.0%)** | 27 (11.3%) | 44 (18.4%) |
| 7 | 15 (6.3%) | 27 (11.3%) | 5 (2.1%) | **39 (16.3%)** | 29 (12.1%) | 36 (15.1%) | **43 (18.0%)** | 45 (18.8%) |
| 8 (least impact) | 17 (7.1%) | 18 (7.5%) | 5 (2.1%) | 36 (15.1%) | **40 (16.7%)** | 37 (15.5%) | 39 (16.3%) | **47 (19.7%)** |

**Bold text** denotes the most frequently assigned rank for each domain.

## **Supplementary Table 3** Caregiver rankings of impact domains

| **Rank** | **Domain (n, %)** | | | | | | | |
| --- | --- | --- | --- | --- | --- | --- | --- | --- |
|  | **Emotional health** | **Financial** | **Physical health** | **Planning and autonomy** | **Occupational** | **Safety** | **Sleep** | **Social** |
| 1 (most impact) | 13 (16.0%) | **30 (37.0%)** | 11 (13.6%) | 4 (4.9%) | 6 (7.4%) | 9 (11.1%) | 5 (6.2%) | 3 (3.7%) |
| 2 | 12 (14.8%) | 8 (9.9%) | **21 (25.9%)** | 11 (13.6%) | 10 (12.3%) | 11 (13.6%) | 6 (7.4%) | 2 (2.5%) |
| 3 | **16 (19.8%)** | 8 (9.9%) | 14 (17.3%) | 11 (13.6%) | **16 (19.8%)** | 2 (2.5%) | 11 (13.6%) | 3 (3.7%) |
| 4 | 9 (11.1%) | 6 (7.4%) | 13 (16.0%) | 7 (8.6%) | 15 (18.5%) | 17 (21.0%) | 8 (9.9%) | 6 (7.4%) |
| 5 | 11 (13.6%) | 7 (8.6%) | 13 (16.0%) | 9 (11.1%) | 9 (11.1%) | 10 (12.3%) | 13 (16.0%) | 9 (11.1%) |
| 6 | 7 (8.6%) | 10 (12.3%) | 5 (6.2%) | 16 (19.8%) | 6 (7.4%) | **17 (21.0%)** | 5 (6.2%) | 15 (18.5%) |
| 7 | 7 (8.6%) | 4 (4.9%) | 2 (2.5%) | **13 (16.0%)** | 10 (12.3%) | 7 (8.6%) | **21 (25.9%)** | **17 (21.0%)** |
| 8 (least impact) | 6 (7.4%) | 8 (9.9%) | 2 (2.5%) | 10 (12.3%) | 9 (11.1%) | 8 (9.9%) | 12 (14.8%) | 26 (32.1%) |

**Bold text** denotes the most frequently assigned rank for each domain.
